# Oral Microbiome in Pre-Rheumatoid Arthritis: The Role of Aggregatibacter Actinomycetemcomitans in Bacterial Composition

**DOI:** 10.7759/cureus.32201

**Published:** 2022-12-05

**Authors:** Arathi Kulkarni, Michelle D Beckler, Sahar S Amini, Marc M Kesselman

**Affiliations:** 1 Osteopathic Medicine, Nova Southeastern University Dr. Kiran C. Patel College of Osteopathic Medicine, Davie, USA; 2 Microbiology and Immunology, Nova Southeastern University Dr. Kiran C. Patel College of Allopathic Medicine, Fort Lauderdale, USA; 3 Internal Medicine, Nova Southeastern University Dr. Kiran C. Patel College of Osteopathic Medicine, Davie, USA; 4 Rheumatology, Nova Southeastern University Dr. Kiran C. Patel College of Osteopathic Medicine, Davie, USA

**Keywords:** anti-citrullinate protein antibody, pre-rheumatoid arthritis, actinobacteria, periodontal disease, rheumatoid arthritis

## Abstract

Rheumatoid arthritis (RA) is a chronic autoimmune disease that symmetrically affects the joints, eventually leading to cartilage and tissue destruction. While there are multiple etiologies for RA, from environmental to genetic risk factors, periodontal disease (PD) may contribute to the acceleration of RA symptoms in pre-rheumatoid arthritis (pre-RA) and RA patients. While PD is caused by multiple oral bacteria, this review explains the role of *Aggregatibacter actinomycetemcomitans (Aa) *in the pathogenesis of pre-RA and RA based on 13 primary articles. This paper focuses on the *Aa* virulence factor leukotoxin A (LtxA) because it has been reported to cause cellular destruction and inflammation in the oral cavity that can accelerate the development of RA. Individuals who are classified as pre-RA may benefit from periodontal screening to further reduce their risk of developing advanced RA. Additionally, they may benefit from earlier pharmacological therapy for RA using disease-modifying anti-rheumatic drugs (DMARD) and antibacterial treatment.

## Introduction and background

Rheumatoid arthritis (RA) is a chronic, progressive, disabling disease affecting up to 1% of the world’s population [1]. RA primarily affects the joints of the hands, knees, and ankles in a symmetric distribution and may also lead to extraarticular symptoms [2]. Laboratory diagnosis can include findings of anti-citrullinated protein antibodies (ACPA) and rheumatoid factor (RF). Both ACPA and RF are self-reactive antibodies that can underlie the immunopathogenesis of RA. However, RA onset and progression are multifactorial, including both genetic and environmental factors. This includes the recent recognition of a link between RA, ACPA, and periodontal disease (PD). Many of the bacteria implicated in PD are components of the oral microbiome. This review focuses on the role of oral bacteria in PD in the pathogenesis of RA and pre-rheumatoid arthritis (pre-RA).

Advanced PD affects 10-15% of the world’s population [3]. Oral bacteria have a commensal relationship in the oral cavity; however, when certain bacteria overgrow in subgingival pockets, the immune system is activated, leading to robust gingival inflammation. Many of the deleterious effects exhibited in PD can be linked to the immune system [4]. In more severe stages, PD can lead to periodontal ligament loss and alveolar bone loss [5]. While PD is considered to have multiple etiologies, anaerobes appear to play a significant role. *Aggregatibacter actinomycetemcomitans* (*Aa*), a commensal bacteria found in the oral flora, may cause opportunistic infections during overgrowth and is strongly associated with localized PD [4]. *Aa* was once shown to be the most commonly isolated bacteria from periodontal lesions, and a study reported that 95% of patients with localized aggressive periodontitis were found to be infected by *Aa* [6]. While there are other oral bacteria found in association with the development of PD such as *Tanerella forsythia, Prevotella intermedia, Treponema denticola,* and *Campylobacter rectus*: *Aa *and *Porphyromonas gingivalis* are two of the more dominant species [7].

When studied along with RA, PD can alter the microbiome and lead to inflammation that predisposes the development of RA in susceptible individuals or further worsens the progression of RA [8]. Both RA and PD increase the presence of proinflammatory cytokines such as IL-1β, IL-6, and TNF-α [6]. *Aa* has several virulence factors, including collagenase (ability to destroy connective tissue), protease (ability to cleave immunoglobulins), endotoxins (lipopolysaccharide), and fibroblast inhibition factors (inducing osteoclastic factors) that all contribute to localized PD disease inflammation [6]. However, one of the most significant virulence factors reported to be linked to RA pathogenesis in the literature is leukotoxin A (LtxA) [4,9,10]. LtxA can cause cellular destruction in neutrophils, granulocytes, monocytes, and some lymphocytes by inducing pores in their cellular membranes [4]. Looh et al. [9] stated that the virulence factor LtxA of *Aa* allows this bacterium to induce inflammation [9]. LtxA may cause hypercitrullination of neutrophils in RA and, thus, allow the bacteria to evade host immune defenses. This leads to persistent infection and inflammation in the host. Additionally, LtxA may kill leukocytes, protecting *Aa* from phagocytosis and other host immune responses [9]. Neutrophils, when exposed to LtxA, degranulate and release proteolytic enzymes and metalloproteases, which contribute to periodontal tissue destruction and create a proteolytic environment that allows for the degradation of immunoglobulins [10]. Looh et al. [9] hypothesized that LtxA-mediated hypercitrullination may involve the abnormal activation of PAD enzymes that lead to the citrullination of proteins and autoantigens recognized by ACPA. These mechanisms can further accelerate the development of RA in susceptible individuals, as patients with RA similarly have an increase in citrullination of proteins and 60-80% of RA patients are ACPA positive [9]. However, it is important to consider other bacteria capable of producing the citrullination seen in RA, as reported in the literature. *P. gingivalis *is another periodontal pathogen linked to citrullination and acceleration of RA [11]. The *P. gingivalis *enzyme peptidylarginine deiminase (PPAD) can convert arginine residues into citrulline, contributing to RA pathogenesis and the formation of ACPA antibodies. Additionally, accelerated cartilage and tissue destruction in RA patients has been seen with the co-infection of *P. gingivalis* [11].

Pre-RA is the preclinical phase of RA in which patients are at risk of developing RA but have not met all diagnostic criteria. Under this designation of pre-RA, patients are not categorized as RA patients and do not fall under the pre-articular phase of RA. Patients classified as pre-RA may test positive for ACPA and/or RF antibodies, but they may also test seronegative for these antibodies [12]. Additionally, abnormalities in immune functioning and mild joint inflammation may be seen before clinically apparent joint stiffness and tissue synovitis in pre-RA [13]. The term pre-RA describes all phases of the disease before diagnostic criteria are met in order to be classified as RA. The European Alliance of Associations for Rheumatology (EULAR) criteria may be used to characterize asymptomatic pre-RA patients as symptomatic pre-RA patients [14].

Prevention of clinically apparent RA is currently undergoing several clinical trials [15] Pharmacological therapies such as hydroxychloroquine, abatacept, and rituximab are being studied for their effects on halting the progression of clinically apparent RA [15]. In a 2017 published study, 81 individuals who were positive for ACPA and RF but without arthritis were either treated with rituximab or a placebo and followed up on average for 29 months. The results showed the time for the development of inflammatory arthritis/RA in 25% of the participants was delayed by an average of 12 months in the rituximab treatment group compared to the control groups [16]. With no cure for RA, it may be beneficial to identify pre-RA patients and remove risk factors that further contribute to RA development and/or begin earlier RA treatment for these individuals. One risk factor of significance is PD, which has a similar pathogenesis to RA. Periodontal treatment may lighten the load of RA in pre-RA patients and prevent disease progression.

## Review

Methods

**Figure 1:**
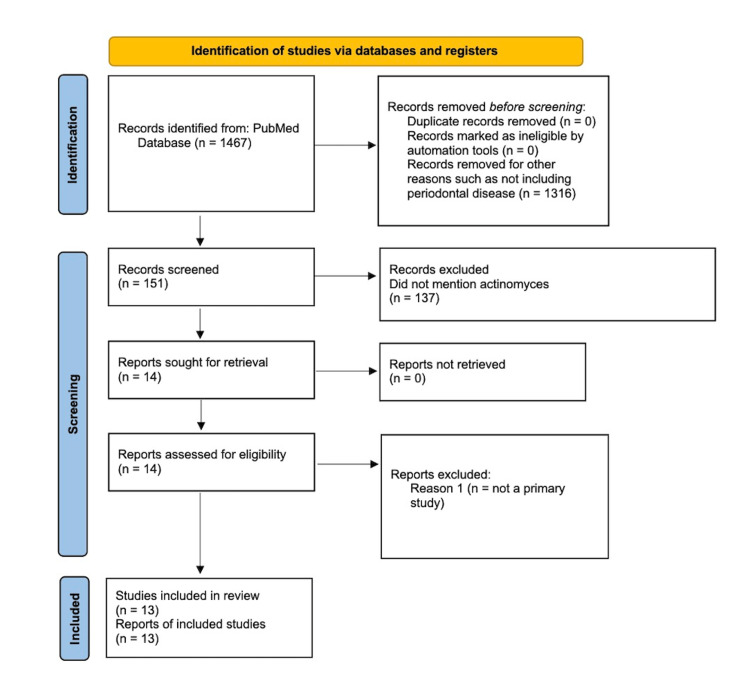
Preferred reporting items for systematic reviews and meta-analyses (PRISMA) chart of the 13 articles included in this review.

For this literature review, all articles were collected from PubMed’s online database, as this review is a simple literature review. The preferred reporting items for systematic reviews and meta-analyses (PRISMA) chart is shown in Figure 1. Only relevant case-control and experimental studies were included in this review. These studies were conducted with animals, humans, or molecular-level models. The keywords used to find relevant articles included RA and PD, RA and periodontal infection, RA and intestinal microbes, Citrullination and *Actinomycetemcomitans, *Periodontitis and RA and Citrullination, Pre-RA and ACPA+, Cyclic citrullinated peptide, Antibodies and development of RA, Precede the symptoms of RA and anti-CCP, and Anti-citrullinated protein antibodies and first-degree relatives of RA. Prior to the screening, 1467 articles were identified. After considering the inclusion and exclusion criteria, 13 articles were selected for this review (Figure 1).

Results

RA and Aa

There are several studies in the literature that describe the relationship between the bacteria *Aa* found in PD and RA. Studies have been done with animals, molecular models, and humans. In a 2018 animal study, three different mouse strains were used to study different oral bacteria’s impact on arthritic development. The three mouse strains included spontaneously developing arthritis, susceptibility to arthritis, and resistance to collagen-induced arthritis. Each strain was subsequently inoculated with either *P. gingivalis, F. nucleatum, Aa*, or a combination of these three bacteria [17]. The results showed that mice inoculated with *Aa *and *F. nucleatum* exhibited periodontitis and significantly accelerated arthritis onset compared to other test and control groups [17]. Findings were significant as this was one of the first animal models to show *Aa* involvement in the progression of arthritis in an animal model. A 2001 study investigated *Aa* on a molecular level to study its association with RA [18]. The researchers found the prevalence of the N terminal region of DNA-J protein in *Aa* is higher in RA patients compared to the control group (p < 0.005) and suggested that this region may have an etiologic role in the development of RA [18]. Human studies evaluating the association between *Aa* presence and RA have also shown a relationship in disease progression. A 2016 case-control study found that gingival crevicular fluid (GCF) from patients with PD was highly enriched in citrullinated proteins similar to the cellular hypercitrullination found in RA joints. Additionally, 47% of RA patients were infected with *Aa* and tested positive for the presence of ACPA. The researchers concluded that *Aa *is one of the only pathogens that could produce the citrillinome found in periodontitis and RA [19]. In a case-control study conducted in 2016, Chen et al. [20] found an increased presence of the phylum *Aa* in the RA group compared to healthy controls (0.45% vs. 0.04%). The researchers specifically found an increased presence of the genera *Eggerthella* and *Actinomyces *from the phylum *Aa* and concluded that this bacterium may have an association with RA pathogenesis, which was consistent with the findings of a 2017 case-control study [21]. In this 2017 published study, the researchers found that *Aa* was detected more often in RA patients and more prevalent in the microbiome of RA patients (chi2, p = 0.012) [21]. Reichert et al. [22] found, in their 2020 published study, all patients infected with *Aa* were simultaneously anti-CCP positive (p = 0.043). They found that *Aa* infection is associated with an increased formation of anti-CCP in RA patients, which may contribute to RA prognosis. These studies further demonstrate the role of *Aa *in RA development in animals, molecular models, and humans. However, there is additional evidence to support that the anti-citrullinated protein found in periodontitis may be independent of *Aa* growth and may be a result of other oral bacteria inoculation.

In their 2018 published study, Engstrom et al. [23] found that increased citrullination seen in periodontitis was independent of the presence of periodontal pathogen *P. gingivalis* and *Aa *leukotoxin. They hypothesized that the citrullination in periodontitis may not be caused by the *Aa* virulence factor LtxA, contradicting several prior studies that hypothesized that the LtxA virulence factor plays a significant role in causing citrullination and further inflammation in RA patients. Additionally, in their 2018 study, Volkov et al. [24] tested whether anti-Ltx Ab was present in RA patients. Their results did not find an association between anti-LtxA Ab and the presence of ACPA or HLA-SE alleles in RA patients. The 2005 published study demonstrated that Aa was not found in significant quantities in RA patients and that additional bacteria were more prevalent in RA patients [25]. The IgG levels of *P. gingivalis*, *P. intermedia*, *P. melaninogenica,* and *B. forsythus* were found to be significantly higher in RA compared with those of the control group, while *Aa* was not found at greater levels in RA serum samples in comparison with the healthy controls.

Pre-RA and Aa

Given the association between the increased presence of *Aa* and RA development, the question following arises: are pre-RA patients more susceptible to RA due to the presence of *Aa*? Patients may be classified as Pre-RA based on the presence of ACPA+ or RF+ without having the clinical signs and symptoms of RA. The 2020 case-control study looked at the *Aa* effect on the development of RA. They studied IgM, IgG, and IgA antibodies with respect to *Aa* LtxA with a cohort of Swedish adults at different stages of RA development [26]. Stages included before the onset of symptoms of RA help establish RA disease. Results showed that patients with early and established RA had increased levels of anti-LtxA IgM compared with matched non-RA controls and periodontally healthy controls. A logistic regression revealed that anti-LtxA IgM levels were associated with RA during early disease (OR 1.012, 95%CI 1.007, 1.017), which was maintained after adjustments were made for smoking, anti-CCP antibodies, rheumatoid factor, HLA-DRB1 shared epitope alleles, and sex [26]. The researchers concluded that there may be a time-sensitive relationship between the exposure of *Aa* in pre-RA asymptomatic patients and RA disease development [26]. Another case-control study published in 2020 found similar results. The researchers studied the presence of *Aa* in 29 pre-RA individuals (positive for ACPA Ab) with no clinical arthritis, 27 RA patients, and 23 healthy controls by examining saliva samples from each cohort [27]. In the pre-RA patient cohort, salivary microbial diversity was reduced compared to patients. Specifically, they found the presence of *Aa* and *Patescibacteria* bacteria was increased in high-risk RA patients [27]. Both studies showed that not only is *Aa* more common in pre-RA patients, but the *Aa* virulence factor may play an important role in the pathogenesis of early RA. However, there is evidence to suggest that *Aa* may not be prevalent in pre-RA patients when compared to other oral bacteria.

Two studies were conducted on oral bacteria’s association with pre-RA patients and demonstrated that *Aa* presence is not elevated in pre-RA patients and, therefore, may not cause accelerated RA development. A 2014 published prospective case-control study compared 22 early-RA (ERA) individuals with 22 matched healthy controls [8]. They found more advanced forms of periodontitis in ERA patients compared to the controls, particularly the bacteria* Streptococcus anginosus* (3.56-fold more, P = 0.028), which is the characteristic pathogen in the supragingival pockets of ERA patients. They concluded *Aa* was not significant in supragingival pockets of the ERA group (0.19-fold, P = 0.049) [8]. A 2019 published study investigated 48 anti-CCP-positive individuals without arthritis, 26 ERA patients, and 32 healthy controls [28]. They found that *P. gingivalis* was found in greater abundance in healthy periodontal sites compared with healthy individuals (effect size, 3.00; 95% CI, 1.71-4.29) and patients with ERA (effect size, 2.14; 95% CI, 0.77-3.52) but did not find a similar association with *Aa* [28]. Both of these studies showed that there may be other oral bacteria found in higher quantities in pre-RA patients when compared to *Aa*.

Discussion

The ability of *Aa* to accelerate RA development in pre-RA patients should be explored further. This review examined 13 articles that studied the relationship between *Aa* presence and its effect on RA, as well as pre-RA pathogenesis. Of the 13 articles, 8 showed an association between *Aa* and RA or pre-RA. These articles demonstrated this by showing the significant presence of *Aa* in these populations and hypothesizing that the *Aa* virulence factor LtxA may play a role in RA pathogenesis. In contrast, five of the eight articles showed that other oral bacteria, namely* P. gingivalis*, may play a more significant role in RA development. *P. gingivalis* has been shown in other studies to have a significant presence in at-risk RA patients. In a 2016 published study, anti-*P. gingivalis (*anti-CPP3) was detected in 5% of pre-symptomatic individuals and 8% of RA patients compared to controls (p < 0.001) [29]. Likewise, other bacteria have also been noted to be present in abundance in pre-RA patients. In a 2014 published study, researchers found the bacteria *T. forsythia* (6.77-fold, P = 0.033) and *S. anginosus* (3.56-fold, P = 0.028) enriched in the subgingival and supragingival pockets, respectively, in ERA patients, suggesting that these bacteria may play a more significant role in aggravating RA in pre-RA patients [8]. Other bacteria, such as *Prevotella* in the saliva and *Veillonella* in the saliva and tongue coating, have also been found in significant quantities in at-risk and ERA individuals, increasing their likelihood of developing RA compared to the healthy controls [30]. While it is important to consider the sampling methods used in these studies, as they could be one of the reasons why certain oral bacteria were found to be more prevalent, the commonality among these studies is that PD may play a significant role in the development of RA in pre-RA patients. Future studies may better elucidate oral bacteria’s relationship with pre-RA patients by lengthening the time course of studies and monitoring any changes in oral bacteria composition throughout the study. The *Aa* virulence factor LtxA should also be further studied to better understand its mechanism for and contribution to accelerating RA pathogenesis. 

A better understanding of the *Aa*’s effect on pre-RA development can help target at-risk pre-RA patients through PD screening to reduce the potential disease burden. PD screening by dentists may be of importance for these at-risk patients for early treatment and reduction in specific oral bacteria. Furthermore, antibacterial and disease-modifying anti-rheumatic drugs (DMARD) therapy can be used in these higher-risk populations to mitigate future disease development.

## Conclusions

There appears to be a relationship between the early pathogenesis of RA and *Aa* found in oral bacteria.* *The *Aa* virulence factor LtxA may play a significant role in the acceleration of RA development in at-risk patients by inducing further citrullination and inflammation. This has been seen in studies on RA patients and pre-RA patients. The relationship between *Aa *and pre-RA can be studied further to risk stratify pre-RA patients in terms of the degree and abundance of bacterial concentration that exists and the resulting inflammation. However, it is important to consider other oral bacteria’s relationship with pre-RA patients, mainly *P. gingivalis* as this bacterium may also play a role in the development of RA. While the relationship between *Aa* and pre-RA needs to be studied further, early interventions through the initiation of pharmacological treatments with DMARDS upon the recognition of positive auto-antibodies prior to the onset of symptoms, dental and periodontal therapies, and the elimination of associated risk factors may be recommended for high-risk pre-RA patients who test positive for *Aa*.
